# Six-month low level chlorine dioxide gas inhalation toxicity study with two-week recovery period in rats

**DOI:** 10.1186/1745-6673-7-2

**Published:** 2012-02-21

**Authors:** Akinori Akamatsu, Cheolsung Lee, Hirofumi Morino, Takanori Miura, Norio Ogata, Takashi Shibata

**Affiliations:** 1 Taiko Pharmaceutical Co., Ltd, Suita-shi, Osaka, Japan; 2 R&D Department, Taiko Pharmaceutical Co., Ltd, 3-34-14 Uchihonmachi, Suita-shi, Osaka 564-0032, JAPAN

**Keywords:** Chlorine dioxide, Gas, Inhalation, Long-term, Toxicity, Whole body

## Abstract

**Background:**

Chlorine dioxide (CD) gas has a potent antimicrobial activity at extremely low concentration and may serve as a new tool for infection control occupationally as well as publicly. However, it remains unknown whether the chronic exposure of CD gas concentration effective against microbes is safe. Therefore, long-term, low concentration CD gas inhalation toxicity was studied in rats as a six-month continuous whole-body exposure followed by a two-week recovery period, so as to prove that the CD gas exposed up to 0.1 ppm (volume ratio) is judged as safe on the basis of a battery of toxicological examinations.

**Methods:**

CD gas at 0.05 ppm or 0.1 ppm for 24 hours/day and 7 days/week was exposed to rats for 6 months under an unrestrained condition with free access to chow and water in a chamber so as to simulate the ordinary lifestyle in human. The control animals were exposed to air only. During the study period, the body weight as well as the food and water consumptions were recorded. After the 6-month exposure and the 2-week recovery period, animals were sacrificed and a battery of toxicological examinations, including biochemistry, hematology, necropsy, organ weights and histopathology, were performed.

**Results:**

Well regulated levels of CD gas were exposed throughout the chamber over the entire study period. No CD gas-related toxicity sign was observed during the whole study period. No significant difference was observed in body weight gain, food and water consumptions, and relative organ weight. In biochemistry and hematology examinations, changes did not appear to be related to CD gas toxicity. In necropsy and histopathology, no CD gas-related toxicity was observed even in expected target respiratory organs.

**Conclusions:**

CD gas up to 0.1 ppm, exceeding the level effective against microbes, exposed to whole body in rats continuously for six months was not toxic, under a condition simulating the conventional lifestyle in human.

## Background

Chlorine dioxide (CD), which is a water-soluble, yellow gas at room temperature, exists as a relatively stable free radical and is a very strong oxidant agent [1-3]. Therefore, when dissolved in water, CD has a potent antimicrobial activity against bacteria and viruses *in vitro *[4-7].

Additionally, recent studies presented that the gas-phase CD also has a potent antimicrobial efficacy [8-10]. In particular, it was reported that the low-concentration CD gas at 0.03 ppm has a protective effect against influenza A virus infection in mice [11]. Also, Ogata and Shibata reported that low-level CD gas-releasing canisters placed in a classroom decreased the absenteeism of schoolchildren in the winter season, presumably because of preventing the occurrence of epidemic cold and influenza [12]. Furthermore, it was shown in a prospective cohort clinical study that the extremely low concentration CD gas, such as 0.01 ppm or 0.02 ppm, prevented against influenza-like illness [13]. Other studies also revealed that the low-concentration CD gas inactivated feline calicivirus (FCV), a norovirus surrogate, which was attached to a glass surface in the wet or dry state [14,15]. The concentrations of CD gas in these studies were not greater than the 8-hour time-weighted average (TWA), which was defined as 0.1 ppm by the US Department of Labor's Occupational Safety and Health Administration (OSHA) [16]. These results suggest that the low-level CD gas may be an effective measure to disinfect space where people live in or gather at, such as houses, offices, airport buildings, and hospitals, as long as the CD gas concentration is not toxic.

However, the toxicity of the low-level, gas-phase CD, particularly the long-term toxicity, has not been well studied. In few inhalation toxicity studies of CD gas, Paulet and Desbrousses [17-19] used high concentration of CD gas, such as 2.5 ppm or higher. Also, the CD gas exposure period was not longer than 45 days. The longest-term study was conducted by Dalhamn [20]. His study showed a no observed adverse effect level (NOAEL) of 0.1 ppm in rats exposed to CD gas for 5 hours/day for 10 weeks. However, because the CD gas concentration during the exposure period fluctuated widely between 0.05 ppm and 0.3 ppm, the value of this study is limited. Therefore, it is currently strongly desired to study whether toxic signs are revealed or not if CD gas, at 0.1 ppm or lower, is exposed to animals for a longer period of time than that in Dalhamn's study. Furthermore, the results of a toxicity study, in which animals are exposed to CD gas continuously for 24 hours, will be useful to simulate the realistic human lifestyle. If safety is confirmed under these conditions, the low-level CD gas may be used continuously for infection control in the environment where human beings are present.

Thus, the purpose of this study was to examine the presence or absence of toxic effects of low-concentration CD gas up to 0.1 ppm, the level of which was precisely regulated when the gas was exposed to rats continuously for a longer term than that of previous studies, under the condition of free access to chow and water, simulating the ordinary human lifestyle. In summary, the rationale for the exposure levels chosen in this study is on the basis of previous studies including ours showing that the antimicrobial effect of CD gas was found from 0.01 ppm [13] to 0.03 ppm [11]. Consequently, we chose the CD gas levels of 0.05 ppm and 0.1 ppm that cover these effective concentrations, still not exceeding 0.1 ppm that is the US OSHA's 8-hour TWA long-term permissible level.

## Methods

### Test animals and housing conditions

Male and female Sprague-Dawley Crl:CD (SD) rats, approximately 5 weeks old, were obtained from Charles River Laboratories Japan (Yokohama, Japan). Two animals of the same gender per cage were housed in animal rooms which were maintained with 24 ± 3°C temperature, 50 ± 10% relative humidity, and 12 hour light/dark cycle. The animals were given free access to CRF-1 diet (Oriental Yeast, Tokyo, Japan) and tap water. They were kept for 1 week for acclimation to the facility and quarantine prior to the start of the exposure period. All procedures were conducted in adherence to the National Institutes of Health Guide for the Care and Use of Laboratory Animals and were approved by the Institutional Animal Care and Use Committee of Hamri Corporation, to which the execution of this study was contracted.

### Generation of CD Gas and inhalation exposure chamber

**Figure 1:**
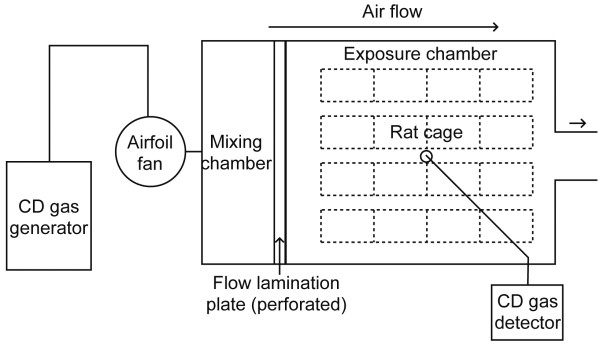
**A schematic diagram of the set-up of a CD gas exposure chamber**. CD gas discharged from the CD gas generator shown at the left was mixed with air by an airfoil fan, then sent to a mixing chamber and flown through a perforated, flow lamination plate to yield an even, regulated concentration CD gas flow throughout cages housing animals in the exposure chamber. A probe of CD gas detector was placed in the middle of the chamber to monitor the CD gas concentration continuously. The ventilation rate in the chamber was 30 times per hour. In order to ensure the even exposure of CD gat to rats, the position of cages were rotated once weekly. In an experiment prior to the study, it was confirmed that the existence of animals, chow, water, and excrements did not affect the flowing CD concentration (data not shown). It was also confirmed that the CD gas concentration was equal at the inlet and at the outlet as well as at the center of the chamber (data not shown).

CD gas was obtained from an electrochemical system [21]. Briefly, CD gas was generated by dissolving the electrolytically-evolved product of potassium chloride into sodium chlorite solution. The experimental set-up for the exposure of CD gas to rats is shown in Figure 1. Hermetic inhalation exposure chambers, each size of which was 700 W × 1350D × 1600H mm, were made up with stainless steel and transparent vinyl chloride plates. The ventilation rate in the chamber was 30 times per hour. To maintain even CD gas flow and homogenous CD gas concentration in the chamber, five small-size (25 mm × 25 mm × 10 mm) direct current electric fans with an airflow rate of 0.048 m^3^/min (F2510CT-12UCV, Shicoh Engineering, Kanagawa, Japan) were placed in each chamber. The rats of the same gender were housed as two animals per stainless steel wire mesh cage in the chamber. The CD gas concentration in the chamber was regulated by adjusting the electric current of the electrolysis, and was monitored continuously by a CD gas detector (CS-7 with a CDS-7 sensor, New Cosmos Electric, Osaka, Japan). The CD gas monitoring device was calibrated once daily against a CD gas glass tube detector (No. 23 M, Gastec, Kanagawa, Japan).

### Experimental design

Three groups, each consisting of 16 male and 16 female rats, were allocated by a stratified random sampling method. Difference in the average body weight of each group fell within ± 20%. These groups were either exposed with CD gas of 0.05 ppm (low), 0.1 ppm (high) or air only (control). The animals were exposed with CD gas for 24 hours per day, 7 days per week for 6 months, except for the time of laboratory animal care (measuring body weight and filling chow and water once weekly, and removing excreta twice weekly) which was conducted within 30 min for each time. In order to eliminate positional differences of CD gas level in the exposure space, cages housing rats were rotated within the chamber once every week. At the end of exposure period, 10 males and 10 females in each group were sacrificed. The remaining 6 male and 6 female animals in each group were maintained for an additional 2-week recovery period during which time they were exposed with only air.

### Observations

During the exposure and recovery period, animals were observed daily for mortality and signs of toxicity. Body weights, as well as food and water consumptions, were recorded once weekly.

### Biochemistry and hematology

Before necropsy, animals were fasted for approximate 16 hours to avoid yielding turbid serum/plasma. Then, animals were euthanized with isoflurane, and blood was drawn from the abdominal aorta. The collected blood was analyzed for aspartate transaminase (AST), alanine aminotransferase (ALT), gamma-glutamyl transpeptidase (γ-GTP), total protein (TP), albumin (ALB), ratio of albumin to globulin (A/G), blood glucose (BG), total cholesterol (TC), triglyceride (TG), blood urea nitrogen (BUN), interleukin-6 (IL-6), tumor necrosis factor-alpha (TNF-α), creatinine (CRE), C-reactive protein (CRP), phosphorus (P), calcium (Ca), sodium (Na), potassium (K), chloride (Cl), prothrombin time (PT), activated partial thromboplastin time (APTT), red blood cell count (RBC), white blood cell count (WBC), hemoglobin concentration (Hb), hematocrit (HTC), platelet count (PLT), mean corpuscular hemoglobin (MCH), neutrophil differential (NE%), eosinophil differential (EO%), basophil differential (BA%), monocyte differential (MO%), and lymphocyte differential (LY%). Bronchoalveolar lavage fluid (BALF) was collected from the left alveolus with 1.0 ml phosphate-buffered saline, and analyzed for the migratory cell count (BC) and the percent of white blood cell (WBC%).

### Necropsy, organ weights and histopathology

After collecting the blood and the BALF, the brain, the hypophysis, the eye, the thyroid, the thymus, the heart, the aorta, the right lung, the trachea, the pharynx, the liver, the spleen, the pancreas, the adrenal gland, the stomach, the kidney, the small intestine, the large intestine, the ovaries, the testes, the bladder, the femur and the nasal cavity were removed from the animals, and were observed macroscopically for signs of the toxicity. After the weight of the brain, the liver, the spleen, the adrenal gland, the ovaries and the testes was measured, all organs were fixed in a 10% formalin solution containing the neutral phosphate-buffered saline. Subsequently, the right lung, the trachea, the pharynx and the nasal cavity were embedded in paraffin, stained with hematoxylin and eosin, and underwent optical microscopy.

### Statistical analysis

All results were expressed as the means ± standard deviation. Two-way repeated-measures ANOVA tests were used to compare the body weight as well as the food and water consumptions of the CD gas-exposed groups with that of the control group. Other parameters were analyzed with Dunnett's test. The level of significance was set at *p *< 0.05 or *p *< 0.01.

## Results

### CD Gas concentrations

**Figure 2:**
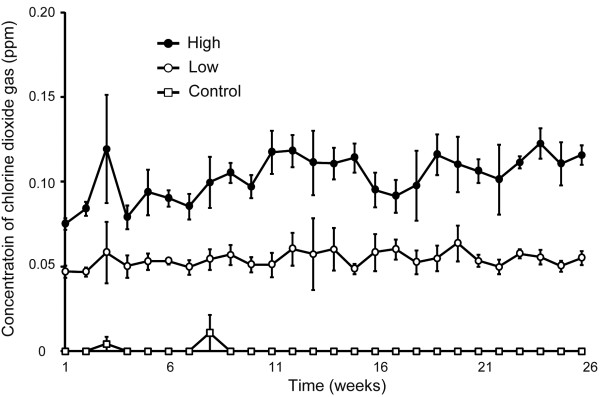
**Time course of changes in the concentration of CD gas in each chamber**. CD gas concentration in each chamber was measured continuously throughout the exposure period for six months. To avoid the Figure looking too busy, symbols show the mean of weekly CD gas concentration (open square: control (air); open circle: 0.05 ppm; closed circle: 0.1 ppm), and error bars represent standard deviations. Throughout the exposure period for six months, there was no tendency of increase or decrease of CD gas concentration, and the fluctuation of the CD gas level was kept within ± 25%, irrespective of the CD dose level. The mean ± standard deviation of CD gas concentration during the exposure period for the low concentration chamber was 0.054 ± 0.007 ppm, and that for the high concentration chamber was 0.103 ± 0.011 ppm.

CD gas concentrations in exposure chambers during the entire exposure period are shown in Figure 2. In the low concentration chamber, the chronological drift of mean CD gas level in each week was between 0.047 ppm and 0.060 ppm, whereas the drift in the high concentration chamber was between 0.075 ppm and 0.120 ppm. The mean ± SD of CD gas concentration during the exposure period for the low concentration chamber was 0.054 ± 0.007 ppm, and that for the high concentration chamber was 0.103 ± 0.011 ppm.

### Macroscopic observation, food and water consumption, and body and relative organ weights

**Figure 3:**
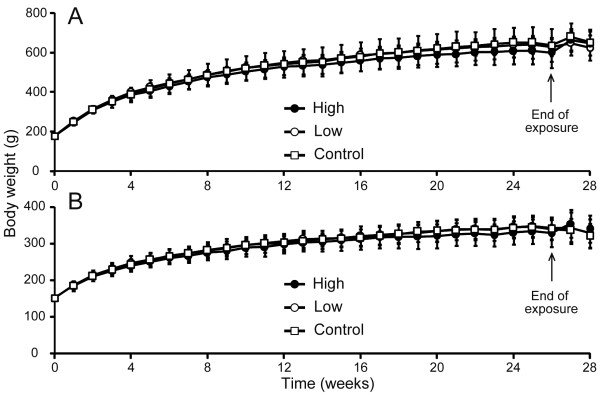
**Body weight changes in rats exposed to CD gas**. Arrows represent the end of the exposure period and the start of the recovery period. (A); Males, and (B); females. Symbols (open square: control; open circle: 0.05 ppm, and closed circle: 0.1 ppm) show the mean body weight and error bars represent the standard deviation. The body weight of animals was measured once weekly. The number of rats for calculating mean ± standard deviations was 16 during the exposure period and 6 during the recovery period. There was no statistically significant change observed between the CD gas-exposed group and the control group throughout the study period.

**Figure 4:**
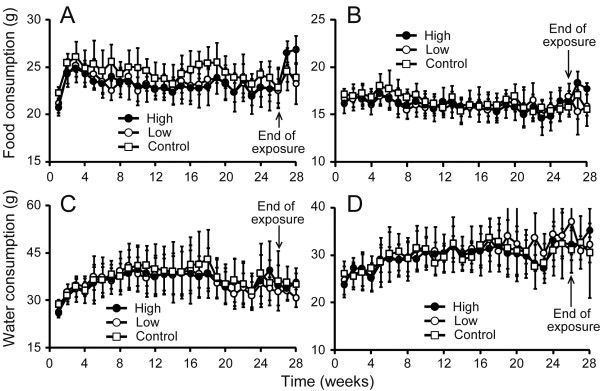
**Food and water consumptions of rats exposed to CD gas**. Arrows represent the end of the exposure period and the start of the recovery period. (A); food consumption in males, (B); food consumption in females, (C); water consumption in males, and (D); water consumption in females. Symbols (open square: control; open circle: 0.05 ppm, and closed circle: 0.1 ppm) show the mean of food and water consumptions and the error bars represent standard deviations. The measurement was performed once weekly. The number of rats for calculating the mean ± standard deviations was 16 during the exposure period and 6 during the recovery period. There was no statistically significant difference between the CD gas-exposed group and the control group throughout the study period, irrespective of the CD gas exposure concentration.

During all study periods, no mortality was observed, and there were no sings of the CD gas-related toxicity in daily observation. There were no statistically-significant changes in the body weight during the exposure period between the exposed and the control groups (*p *= 0.348 in male, *p *= 0.466 in female; Figure 3). Furthermore, no statistically significant differences were observed in food and water consumptions between the exposed and the control rats (food consumption: *p *= 0.101 in male, *p *= 0.613 in female; water consumption: *p *= 0.848 in male, *p *= 0.812 in female; Figure 4). With respect to the relative organ weight, no statistically significant changes were observed in male and female rats during the exposure period (Table 1). Additionally, during the recovery period, no significant relative organ weight changes were observed either in male or female rats (data not shown).

**Table 1:** Relative Organ Weight (%) of Rats Exposed to CD Gas for 6 Months

Group	Control		Low		High	
**Organ**	**Male**	**Female**	**Male**	**Female**	**Male**	**Female**

Brain	0.36 ± 0.05	0.60 ± 0.06	0.34 ± 0.02	0.59 ± 0.04	0.38 ± 0.06	0.64 ± 0.07

Liver	2.50 ± 0.20	2.46 ± 0.19	2.43 ± 0.28	2.47 ± 0.15	2.47 ± 0.15	2.43 ± 0.26

Spleen	0.13 ± 0.01	0.17 ± 0.02	0.13 ± 0.01	0.16 ± 0.01	0.13 ± 0.01	0.16 ± 0.03

Adrenal gland (right)	0.01 ± 0.00	0.01 ± 0.00	0.01 ± 0.00	0.01 ± 0.00	0.01 ± 0.00	0.01 ± 0.00

Adrenal gland (left)	0.01 ± 0.00	0.01 ± 0.00	0.01 ± 0.00	0.01 ± 0.00	0.01 ± 0.00	0.01 ± 0.00

Testis (right)	0.29 ± 0.04		0.28 ± 0.05		0.32 ± 0.04	

Testis (left)	0.30 ± 0.04		0.30 ± 0.02		0.32 ± 0.05	

Ovary (right)		0.02 ± 0.01		0.01 ± 0.01		0.02 ± 0.01

Ovary (left)		0.02 ± 0.00		0.01 ± 0.01		0.02 ± 0.01

Each value represents mean ± standard deviation of 10 rats

### Biochemistry and hematology values

Some parameters in biochemistry and hematology tests during the exposure period resulted in statistically significant differences between the CD gas-exposed and the control rats (Tables 2 and 3). Among these parameters, the changes in Ca and MO% for male rats and BC for female rats were not dose-related, as the high dose exposed group did not show a statistically significant change. Although EO% obtained either from high or low exposure male rats was significantly lower than those obtained from the control rats, it was also not a dose-related change. In the recovery period, no parameter observed was attributable to CD gas-related changes (data not shown). There was a statistically significant change in BUN in the high exposure female rats.

**Table 2:** Biochemistry Values of Rats Exposed to CD gas for 6 Months

Group	Control		Low		High	
**Analyte**	**Male**	**Female**	**Male**	**Female**	**Male**	**Female**

AST (U/l)	115.3 ± 34.7	140.6 ± 64.3	99.6 ± 20.0	115.5 ± 21.8	99.7 ± 22.1	179.6 ± 178.5

ALT (U/l)	37.5 ± 10.5	55.7 ± 39.6	33.4 ± 7.5	38.1 ± 11.1	38.8 ± 15.7	82.8 ± 116.4

γ-GTP (U/l)	1.0 ± 0.0	1.1 ± 0.3	1.0 ± 0.0	1.0 ± 0.0	1.0 ± 0.0	1.0 ± 0.0

TP (g/dl)	6.2 ± 0.2	7.3 ± 0.6	6.0 ± 0.4	7.3 ± 0.4	6.2 ± 0.2	7.2 ± 0.6

ALB (g/dl)	2.3 ± 0.1	3.0 ± 0.3	2.2 ± 0.1	3.1 ± 0.2	2.3 ± 0.1	3.1 ± 0.3

A/G	0.58 ± 0.03	0.72 ± 0.07	0.58 ± 0.04	0.74 ± 0.04	0.60 ± 0.04	0.76 ± 0.06

BG (mg/dl)	137.4 ± 11.3	137.1 ± 24.6	135.3 ± 27.6	131.2 ± 14.7	132.7 ± 13.0	129.2 ± 16.3

TC (mg/dl)	75.2 ± 14.2	87.5 ± 26.2	67.3 ± 18.2	101.6 ± 23.0	76.2 ± 16.0	88.5 ± 29.3

TG (mg/dl)	71.0 ± 19.4	84.4 ± 50.1	61.1 ± 21.9	118.6 ± 65.7	64.9 ± 32.9	69.1 ± 36.0

BUN (mg/dl)	14.1 ± 2.1	13.1 ± 1.6	14.0 ± 1.9	13.6 ± 2.3	15.4 ± 2.1	15.2 ± 0.9*

CRE (mg/dl)	0.33 ± 0.03	0.38 ± 0.05	0.33 ± 0.05	0.35 ± 0.02	0.36 ± 0.03	0.36 ± 0.02

CRP (mg/dl)	0.03 ± 0.00	0.03 ± 0.00	0.03 ± 0.00	0.03 ± 0.00	0.03 ± 0.00	0.03 ± 0.00

P (mg/l)	6.3 ± 0.4	5.8 ± 0.4	6.2 ± 0.5	5.3 ± 0.7	6.2 ± 0.4	6.1 ± 0.8

Ca (mg/l)	10.3 ± 0.31	1.2 ± 0.6	9.9 ± 0.2*	11.0 ± 0.2	10.3 ± 0.3	11.2 ± 0.6

Na (mEq/l)	143.6 ± 1.0	141.6 ± 1.5	143.5 ± 1.5	141.5 ± 1.5	143.7 ± 0.9	142.0 ± 1.2

K (mEq/l)	4.6 ± 0.2	4.8 ± 0.6	4.7 ± 0.2	4.4 ± 0.3	4.6 ± 0.2	4.6 ± 0.6

Cl (mEq/l)	104.6 ± 1.4	102.6 ± 2.4	105.1 ± 0.7	102.2 ± 1.2	103.9 ± 1.7	102.1 ± 2.0

PT (sec)	15.9 ± 2.0	10.6 ± 0.6	15.0 ± 1.6	10.7 ± 0.7	15.6 ± 1.7	11.6 ± 3.2

APTT (sec)	22.7 ± 3.1	24.9 ± 6.0	20.6 ± 2.1	22.5 ± 3.1	21.1 ± 2.3	24.0 ± 12.5

IL-6 (pg/ml)	ND	ND	ND	ND	63.8 ± 24.1	ND

TNF-α (pg/ml)	ND	ND	ND	ND	ND	ND

Each value represents mean ± standard deviation of 10 rats

* Significantly different from control, *p *< 0.05

ND: Not detected (below the lower quantification limit: TNF-α; < 12.5 pg/ml, IL-6; < 62.5 pg/ml)

**Table 3:** Hematology and BALF Values of Rats Exposed to CD Gas for 6 Months

Group	Control		Low		High	
**Analyte**	**Male**	**Female**	**Male**	**Female**	**Male**	**Female**

**Blood Cell Counts**						

RBC (10^2^/μl)	1108 ± 85	942 ± 124	1039 ± 111	834 ± 72	1019 ± 117	881 ± 130

WBC (10^4^/μl)	89 ± 20	62 ± 15	94 ± 29	56 ± 18	92 ± 21	63 ± 22

Hb (g/dl)	18.1 ± 1.4	16.8 ± 1.8	17.5 ± 2.2	15.2 ± 1.3	17.1 ± 2.0	16.8 ± 2.6

HTC (%)	54.4 ± 3.8	49.5 ± 5.9	52.1 ± 6.6	44.6 ± 4.0	51.7 ± 6.4	47.3 ± 7.5

PLT (10^4^/μl)	78.4 ± 18.0	92.4 ± 18.7	85.5 ± 20.9	90.9 ± 18.7	93.2 ± 31.2	81.2 ± 19.5

MCH (pg)	16.3 ± 0.4	17.9 ± 0.6	16.8 ± 0.6	18.3 ± 0.8	16.8 ± 0.7	18.3 ± 0.9

NE% (%)	14.9 ± 5.8	12.5 ± 6.5	10.4 ± 3.1	13.7 ± 3.5	17.1 ± 4.8	17.5 ± 6.3

EO% (%)	0.9 ± 0.6	1.2 ± 0.9	0.2 ± 0.4*	0.7 ± 0.6	0.3 ± 0.3*	1.1 ± 0.6

BA% (%)	0.5 ± 0.6	0.5 ± 0.5	0.5 ± 0.4	0.8 ± 0.3	0.5 ± 0.	30.2 ± 0.3

MO% (%)	12.2 ± 4.2	12.5 ± 3.5	8.8 ± 2.4*	13.7 ± 3.0	11.5 ± 4.8	13.1 ± 6.3

LY% (%)	69.3 ± 7.9	71.9 ± 6.6	75.4 ± 5.3	67.9 ± 6.3	68.5 ± 4.8	66.2 ± 8.9

**BALF Cell Counts**						

BC (10^4^/ml)	39.4 ± 45.1	12.5 ± 10.0	27.1 ± 37.6	39.9 ± 18.1*	21.8 ± 13.1	28.4 ± 23.5

WBC% (%)	0.0 ± 0.0	0.0 ± 0.0	0.0 ± 0.0	0.0 ± 0.0	0.0 ± 0.0	0.0 ± 0.0

Each value represents mean ± standard deviation of 10 rats

* Significantly different from control, *p *< 0.05

### Necropsy and histopathology

No signs of the toxicity were observed in all organs on macroscopic necropsy examination. On the histopathological observation of putative target organs, such as the nasal meatus, the nasal cavity, the pharynx, the trachea and the bronchus, no aberrant findings attributable to inflammation, such as mucosal asthenia, infiltration and increase in the amebocytes, and increase in the folliculus lymphaticus, were observed (data not shown). We observed a large amount of hemosiderin in blood vessels after the exposure period, and it was deposited to the submucosa of pulmonary capillaries. However, since this finding was observed both in the exposed group and the control group, it was not attributable to the CD gas exposure. Also, during the recovery period, no changes between the exposed and the control group were observed (data not shown). Macrophage propagation, although it was mild, was found in a few rats during the exposure period as well as the recovery period. However, because the propagation was also observed in a few female control rats during the recovery period, the finding was thought to be a common phenomenon. An increase in amebocytes was observed in a few rats both in the exposed and control group, therefore the finding was not regarded as CD gas exposure-related.

## Discussion

The results of our study demonstrate that there was no significant toxicity of low-level CD gas, not greater than 0.1 ppm, following 24 hours/day, 7 days/week and 6-months whole body exposure in rats. This exposure period was longer than that of other previously reported studies [17-20]. Additionally, we successfully regulated the CD gas concentration within ± 25% of the target concentration throughout the exposure period, in comparison with Dalhamn's study in which actual CD gas levels fluctuated widely between 0.05 ppm and 0.3 ppm against the target concentration of 0.1 ppm [20]. According to the US OSHA, the long-time (8 hour) permissible exposure TWA level of CD gas in environmental air in a human workplace is 0.1 ppm. Our results support that this level is not toxic in rats, even if the exposure was continued for 24 hours. Also, in an experiment before the study, we confirmed that the existence of animals, chow, water and excreta did not affect the circulating CD gas concentration under our experimental condition, presumably because of the high cycle time of ventilation, *i.e*., 30 times per hour (data not shown). Moreover, we confirmed prior to the study that the CD gas level was equal in the chamber and in the cage, irrespective of where in the chamber it was measured.

Because of the inhalation exposure of the oxidant agent, it was expected that the primary target organs would be the respiratory organs. However, there were no significant changes of WBC in the blood and the BALF. Also, the cytokines (TNF-α and IL-6), which increase in the presence of inflammation [22,23], were almost not detectable (Table 2), below the lower quantification limit (TNF-α: < 12.5 pg/ml; IL-6: < 62.5 pg/ml). In the high exposure group of male rats, IL-6 was detectable (63.8 pg/ml). However, this value was near the lower quantification limit level. Additionally, in the histopathological observation, no signs of toxicity in the nasal meatus, the nasal cavity, the pharynx, the trachea and the bronchus were observed. Thus, we concluded that the whole body exposure of CD gas, not greater than 0.1 ppm for 24 hours/day, 7 days/week for 6 months, did not cause the inflammatory sign in the respiratory organs in rats.

In biochemistry and hematology examinations, we observed that some parameters showed statistically significant changes. However, almost all of the changes in these parameters were not dose-related. EO%, which was reported to increase as a result of allergy, such as asthma or infection of parasitic arthropods [24-27], showed a statistically significant change between the exposed and the control groups. However, it was not a dose-dependent change, and decreased in the exposed group compared with the control group. To our speculation, since the presumable mechanism of action of CD is oxidation that is a physicochemical reaction, it's toxicity, if any, should appear in a dose-dependent manner. Thus, we concluded that the statistically significant change of EO% was not a sign of CD gas-related toxicity. There was a statistically significant change in BUN in the high exposure female rats. However, according to a report of Charles River Laboratories Japan for SD rats [28], mean ± standard deviation of BUN value in 30-week old SD rats were 16.9 ± 1.9 mg/dl. Also, the mean values in the control and low exposure groups were lower than the reference range. Thus, we concluded that the BUN value in the high exposure female rats was within the normal range and was not a toxic sign.

In previous studies, LOAEL of 2.5 ppm for 30 days' exposure [17], LOAEL of 1 ppm for two months [18], NOAEL of 5 ppm and LOAEL of 10 ppm for 4 weeks [19], and NOAEL of 0.1 ppm for 10 weeks and LOAEL of 10 ppm for 2 weeks [20], all studied in rats, were reported. However, since our primary objective in this study was to reveal that the CD gas up to 0.1 ppm (the concentration that exceeds the effective level against microbes) was safe, even after the longest period of exposure time. Therefore, although we should admit that we were unable to determine the ordinary NOAEL and/or LOAEL levels, our study was still valuable in terms of confirming that the CD gas concentration that was effective against microbes was not toxic.

It was reported that CD gas at a low concentration prevented an infectious respiratory disease *in vivo*, and also inactivated feline calicivirus, influenza A virus, *Staphylococcus aureus *and *Escherichia coli in vitro *[11-15]. According to these studies, effectiveness of CD gas was shown below 0.1 ppm. Hence, our study results suggest that the protective level of CD gas against infectious agents may not be a toxic level in rats, therefore the continuous exposure of a low level CD gas not greater than 0.1 ppm may have a possibility to be an effective measure to disinfect spaces where people live in or gather at, such as houses, offices, airport buildings and hospitals, although some uncertainty factors, such as inter-species differences and inter-individual variations, should be taken into account. Furthermore, a higher dose(s) may have to be employed so as to define the LOAEL as well as NOAEL. Also, additional toxicity studies, such as a developmental toxicity study and a reproduction toxicity study, will be necessary to warrant the safety of continuous exposure of low-level CD gas in public space for the purpose of controlling infection from various, airborne and surface-residing microorganisms.

## Abbreviations

CD: Chlorine dioxide; AST: Aspartate transaminase; ALT: Alanine aminotransferase; γ-GTP: Gamma-glutamyl transpeptidase; TP: Total protein; ALB: Albumin; A/G: Ratio of albumin to globulin; BG: Blood glucose; TC: Total cholesterol; TG: Triglyceride; BUN: Blood urea nitrogen; IL-6: Interleukin-6; TNF-α: Tumor necrosis factor-alpha; CRE: Creatinine; CRP: C-reactive protein; P: Phosphorus; Ca: Calcium; Na: Sodium; K: Potassium; Cl: Chloride; PT: Prothrombin time; APTT: Activated partial thromboplastin time; RBC: Red blood cell count; WBC: White blood cell count; Hb: Hemoglobin concentration; HTC: Hematocrit; PLT: Platelet count; MCH: Mean corpuscular hemoglobin; NE%: Neutrophil differential; EO%: Eosinophil differential; BA%: Basophil differential; MO%: Monocyte differential; LY%: Lymphocyte differential; BALF: Bronchoalveolar lavage fluid; BC: Migratory cell count in BALF; WBC%: Percent of white blood cell in BALF; LOAEL: Lowest observed adverse effect level; NOAEL: No observed adverse effect level.

## Competing interests

The authors declare that they have no competing interests.

## Authors' contributions

AA translated, edited, and finalized the manuscript; CL supervised the entire study; HM contributed to the design of the study; TM was involved in the conception and design of the study; NO contributed to critical review of the study design and the manuscript; TS contributed to the final approval of manuscript. All authors read and approved the final manuscript.
